# Understanding Marital Rape Perception in MENA and South Asia: The Role of Resistance, Sexual Deprivation, and Jealousy

**DOI:** 10.1177/10778012251366222

**Published:** 2025-08-11

**Authors:** Olga Khokhlova, Miranda Horvath, Katherine Allen, Nishtha Lamba

**Affiliations:** 1 Institute for Social Justice and Crime, 102099 University of Suffolk, Ipswich, UK; 2 Psychology Department, Murdoch University Dubai, Dubai, UAE; 3 Psychology Department, 156575 Middlesex University Dubai, Dubai, UAE

**Keywords:** sexual violence, intimate partner violence, underrepresented samples, domestic violence, family violence

## Abstract

Exploring perceptions of marital rape (MR) across diverse regions, including the Middle East and North Africa (MENA) and South Asia, this study recruited participants (*N* = 576, *M*_age _= 24.88, *SD*_age_ = 9.36) via convenience and snowball sampling for online surveys. Participants engaged with nine MR scenario vignettes, varying in perpetrator justification (no justification, sexual deprivation, jealousy) and victim resistance (passive, verbal, verbal, and physical). Results from nine 3 × 3 factorial ANOVAs revealed significant effects of justification type and resistance level on perception of MR. Cultural differences emerged, with MENA respondents emphasizing jealousy-justified rape seriousness, while South Asian respondents were influenced by resistance level. Understanding these regional nuances is crucial for tailored interventions and legal reforms addressing MR.

## Introduction

Marital rape (MR) is a serious societal issue and public health problem that has received limited attention. In most cultures, MR remains unacknowledged or misunderstood. Beliefs related to “sanctity” of marriage (Torres, 2016), assumed right of a husband over his wife (Susila, 2013), and unclear rules related to sexual consent of any kind, not only consent in marriage (Gruber, 2016), are a few factors which contribute to vagueness in interpretation of MR (Mahoney & Williams, 1998). Experiences and narratives of sexual violence, especially in the institution of marriage, were historically silenced and not recognized as a social and public issue. In fact, the first published peer-reviewed paper on the topic of MR did not appear until the late 1970s (Gelles, 1977). However, the literature from the women's suffrage era, which primarily spans the late 19th and early 20th centuries, often addressed issues related to women's rights, domestic violence, and the unequal power dynamics within marriages. While the explicit term “marital rape” might not have been commonly used during that time, discussions on what we now understand as MR or intimate partner violence were present in some writings. Authors and activists from the women's suffrage movement highlighted the lack of legal protection for women within marriages, shedding light on the pervasive issue of spousal abuse. They challenged societal norms that condoned violence against women, advocated for legal reforms to address these injustices and touched on the broader theme of women's autonomy within marriage, including the right to control their bodies and consent to intimate relations (Gilman, 1981; Stanton, 1974).

This study has two main aims. First, to examine the impact of a number of factors that have so far received limited attention in the literature on MR. Secondly, to broaden the geography of research on the topic and to look not only at how MR is perceived in the Western world but also in regions traditionally less studied, such as the Middle East and North Africa (MENA) and South Asia. The latter seems very important as, according to a 2008 survey of the top psychology journals found that 96% of subjects were from Western, educated, industrialized, rich, and democratic (WEIRD) societies, which reduces the possibility of generalizing such data, as only 12% of the world's population live in Western industrialized countries (Henrich et al., 2010). In the context of a complex issue like MR, this imbalance means that the experiences of the majority of women remain unnoticed or at least unaddressed.

Furthermore, even acknowledging that most people are not WEIRD represents a kind of generalization because it places 88% of the world's population in the same group. To avoid such generalization, it is necessary to compare participants’ responses from the two regions to understand their specificities more fully. This comparison seems appropriate because family rape in both regions has a similar legal status. Namely, it is not criminalized in the vast majority of countries. Understanding the specificities of different regions is essential to offer tailored interventions that are clear to the people of a particular culture and, therefore, adequate and effective.

It seems that the greatest interest of Western researchers in the topic of MR occurred in the 1980s and 1990s when the fundamental works by Russell (1990) and Finkelhor and Yllo (1985) were written. This time coincided with a breakthrough in the conceptualization of this type of violence and its criminalization in the United States, Europe, and other Western countries. Important reviews were also written in 2007 by Martin et al. and in 2003 by Bennice and Resick. Since then, however, the interest of Western researchers in the subject seems to have waned somewhat. MR is undeniably still being discussed in Western countries (Yllö & Torres, 2016), but much less frequently, often in the broader context of either sexual violence or domestic violence. At the same time, there appears to be increasing debate about MR in countries with greater gender inequality. For example, there is a growing interest from Indian authors, who have published several important papers on the topic recently (e.g., Agarwal et al., 2022; Banerjee & Rao, 2022; Damania & Singh, 2022; Deosthali et al., 2022; Rao et al., 2022). It seems not coincidental that the boom in these works comes at a time when there is a growing debate in Indian society about the legal status of MR. This trend is visible not only in India but also among other researchers working in the South Asian context (e.g., Ali et al., 2020; Gautam & Jeong, 2019; Saleem, 2021). However, there are almost no works on MR written in the MENA; this topic is still taboo on a social and legal level, and, perhaps as a consequence, public interest in it has not yet been articulated.

The best available estimates refer to the American population and suggest that between 10% and 14% of married or cohabitating women have been raped at least once by their partner (Finkelhor & Yllo, 1985; Russell, 1990). Among ever-married women, intimate partner (husbands and ex-husbands) perpetrated rape was 4 times more common than a stranger rape (Finkelhor & Yllo, 1985; Russell, 1990), and most women who experience rape by a partner are highly likely to get raped multiple times (Mahoney, 1999). These studies only looked at the American population, and it is arguable how generalizable these findings are. For example, recent studies conducted in the South Asian context suggest that this crime is committed 40 times more in comparison to rape by a stranger (Gupta, 2014); some claim that as many as one in every three men rape their wives in developing countries such as India (Kamdar, 2020). However, despite these alarming statistics, MR has received limited attention and has been largely overlooked not only in the rape but also in domestic violence literature (Bennice & Resick, 2003).

It is argued (Mahoney & Williams, 1998) that this tradition of failing to recognize wife rape as a problem reflects cultural beliefs about relationships, men, women, and sexuality. Beliefs such as the idea that women's sexuality can be owned by her husband and men are entitled to have sexual relationships with their wives are still very prevalent. In addition to this, the veil of silence about sexual relationships between partners, which implies that what happens between husband and wife in the bedroom is a private matter, makes it very difficult to openly discuss this problem (Mahoney & Williams, 1998). Studies show that, despite a greater likelihood of occurrence (Finkelhor & Yllo, 1985), MR is less likely to be recorded as a “rape” than stranger or acquaintance rape (Kirkwood & Cecil, 2001) or considered as a serious crime (Monson et al., 1996, 2000). One possible explanation for this failure to conceptualize MR as a rape is that majority of the victims of MR are unable to conceptualize their experience as rape and therefore could not recognize themselves as “victims” due to the widespread ideas such as “wifely obligation,” “victim blame (VB),” and “social disgrace” associated with MR (Basile, 2002). In addition to that, MR victims are more likely to be blamed than perpetrators (Monson et al., 1996, 2000). Victim-blaming is a general problem with all types of rape, because rape mythology reinforces rape, and defines it too narrowly, by using a definition that excludes the experiences of many women (Peterson & Meuhlenhard, 2004). Moreover, marital status itself can have an impact on the level of rape myth adherence. For instance, it was found that people are less likely to support rape myths when a couple is separated or legally divorced (Ewoldt et al., 2000).

Studies dedicated to rape myths in general have found many factors that affect perception of rape and responsibility attribution in stranger and acquaintance rape (Angelone et al., 2015, Gravelin et al., 2019). For instance, the credibility of the victim is largely impacted by her alcohol consumption and the level of intoxication when the rape took place (Horvath & Brown, 2006; Landström et al., 2016; Romero-Sánchez et al., 2012). Another factor that leads to greater victim blaming (VB) is her perceived promiscuity (Gray & Horvath, 2018; Ramey, 2023) or her level of resistance (Black & McCloskey, 2013; Cohn et al., 2009). However, marital, stranger, and acquaintance rape may differ due to the distinct dynamics involved in each type of sexual assault (Gravelin et al., 2019). Therefore, the most examined factors in sexual aggression might not play such an important role in the perception of MR, and it seems to be a critical area of research to uncover and examine factors that influence victim-blaming and perpetrator support regarding MR.

Previous research identified that the perceived level of resistance might be an important factor to look at since victims who resist MR often employ verbal means of resistance. Most MR victims are either unable or afraid to resist sexual aggression by their husbands. The possible reason for the differences in the level of resistance between MR and stranger rape might be the chronic nature of the event and a resulting learned helplessness (Finkelhor, 1987). If a person experiences ongoing abuse within their marital relationship, they may believe that their efforts to resist or escape the abuse are futile. Over time, they may develop a sense of helplessness, feeling trapped and unable to change their circumstances and seeing no reason to continue to fight for themselves (Goodmark, 2020). At the same time, it should be acknowledged that calling this response learned helplessness might be a biased way to conceptualize what is going on with the victim because, on some occasions, feeling helpless might be an accurate perception of the marital situation and prospects for safety. Previous research suggests that the presence of force and/or coercion has been identified as an essential component of the rape determination process (Koss et al., 1988); in other words, the general audience, as well as victims and potential jurors may fail to recognize the event as a rape if there was no resistance. This seeming absence of resistance might be one of the reasons for MR to be perceived as less severe since lower levels of VB are usually associated with verbal or physical resistance relative to when no overt resistance is displayed (Cohn et al., 2009; Ryckman et al., 1992). However, this variable needs further investigation because research findings are inconsistent; for instance, Deitz et al. (1984) found that participants were more positive toward the perpetrator and viewed him as less responsible for the rape when the victim physically resisted or was unable to resist relative to the verbally resistant victim.

In line with research that found victim's perceived promiscuity to be an important contributor to VB and jealousy correlating with abuse (Lau & Davis, 2003) and being predictive of expressed violence (Bookwala et al., 1992) it could be assumed that in the situation where infidelity is implied, observers attributed greater victim culpability and lower perpetrator criminality and the rape itself will be perceived as less severe. Jealousy and possessiveness within a marriage can create an environment where one partner feels entitled to sexual access to the other, even against their will. These feelings of jealousy can be used to rationalize nonconsensual sexual acts. By studying the role of jealousy and possessiveness in the perception of MR, we can uncover the underlying emotional and psychological factors that contribute to this form of abuse. In addition to that, the existing beliefs about “marital duty” and husbands owning their wives’ sexuality (Mahoney & Williams, 1998) could lead to greater willingness to justify MR in the cases where the wife is perceived as having failed to do her marital duties and the husband is “sexually deprived” because of that. Thus, sexual deprivation, resulting from a partner's choosing not to engage in sexual activities, can lead to a complex power dynamic within a marriage. It is essential to investigate how sexual deprivation can be used as a form of control or manipulation, contributing to the perception of MR. This examination sheds light on the broader issue of spousal control and emotional abuse that often accompanies sexual deprivation.

Thus, this article will focus on the examination of the level of resistance, sexual deprivation, and jealousy as factors contributing to the perception of MR, because their role is essential for a comprehensive understanding of this complex issue. These factors intersect with societal norms, power dynamics, and legal interpretations, shaping how MR is perceived, experienced, and addressed. It is hypothesized that VB and rape support (RS) will be greater when resistance is passive in comparison to verbal or verbal and physical resistance. In terms of different types of justification, it is quite difficult to predict in advance which kind of justification will result in more VB: sexual deprivation or jealousy, and we are likely to see regional differences. In terms of cross-cultural comparisons, it is hypothesized that South Asian participants will be less likely to blame the victim and support rape than MENA participants. Because even though a large number of countries in both of these regions have not yet criminalized domestic rape, except for Bhutan and Nepal in South Asia and Israel, Tunisia, and Turkey in the MENA region, the situation seems to be somewhat more tense in MENA. Because even now, many countries in the Middle East (e.g., Libya, Kuwait, Iraq, Bahrain) have so-called rape-marriage laws in their constitutions and penal codes. These laws have led to impunity for rapists and trapped women and girls in unwanted, abusive marriages (Kheetan, 2017).

## Methodology

### Participants

A sample of 576 participants (*M*_age_ = 24.88, *SD*_age_ = 9.36, age range = 18–69) were recruited through the combination of convenience and snowball sampling. Forced responses were not used, and only participants who decided to submit their answers at the end were analyzed. Convenience sampling was considered suitable because it saves time, allows easy accessibility, and is cost-effective (Bornstein et al., 2013). Snowball sampling was necessary because MENA communities are somewhat closed with limited access to outsiders, and previous literature suggests snowball sampling techniques are advisable for such communities (Cohen & Arieli, 2011; Valerio et al., 2016). After cleaning the data, nine participants were excluded from the final sample as they were younger than 18 years old. As one of the aims was to see cross-cultural differences, after calculating RS, VB and perceived seriousness (PS) of rape in the overall sample, we have only tested participants from MENA and South Asia, excluding participants from North America, Western and Eastern Europe, and Africa (excluding North African communities belonging to MENA), (*n* = 116).

Thus, the final sample for the cross-cultural analysis comprised 451 participants (*M*_age_ = 24.59, *SD*_age_ = 9.66, age range = 18–69). Of these, 63.90% were female (*n* = 288), 33.50% were male (*n* = 151), 0.70% (*n* = 3) preferred not to reveal their gender, and 2.00% (*n* = 9) have provided specified other genders (e.g., nonbinary, genderfluid, transgender, agender, and genderqueer). This final sample consisted of 167 participants from the MENA region (*M*_age_ = 24.46, *SD*_age_ = 8.85, age range = 18–60) and 284 from South Asia (*M*_age_ = 24.09, *SD*_age_ = 10.08, age range = 18–69). 80.60% of participants from South Asia have chosen India as their country of origin (*n* = 229), 13.00% said that they were born in Pakistan (*n* = 37), and the remaining 6.4% (*n* = 18) were born in Sri Lanka, Afghanistan, Bangladesh, and Nepal. The geography of participants from the MENA region was more diverse (see Table 1).

**Table 1.** Country of Origin of Participants From MENA Region.

	Country of origin	*N*	%
1	UAE	51	30.5
2	Jordan	19	11.4
3	Iran	17	10.2
4	Iraq	11	6.6
5	Egypt	8	4.8
6	Palestine	8	4.8
7	Saudi Arabia	7	4.2
8	Lebanon	7	4.2
9	Tunisia	7	4.2
10	Algeria	6	3.6
11	Kuwait	6	3.6
12	Yemen	6	3.6
13	Sudan	4	2.4
14	Syria	4	2.4
15	Qatar	2	1.2
16	Bahrain	2	1.2
17	Oman	1	.6

*Note.* MENA = Middle East and North Africa.

The MENA subsample contained a relatively similar number of women (53.30%, *n* = 89) and men (45.50%, *n* = 76), whereas the South Asian sample consisted of more women (70.10%, *n* = 199) than men (26.40%, *n* = 75).

The participants had diverse levels of education, consisting of individuals with Incomplete School Education (2.50%), Completed School Education (32.80%), Professional Qualification (3.7%), Bachelor's degree (48.60%), Master's degree (10.60%), and a PhD (1.80%; for a regional breakdown, see Table 2).

**Table 2.** Level of Education of Participants From South Asia and MENA Region.

	South Asia (*n* = 284)	MENA region (*n* = 167)
Incomplete school education	2 (0.70%)	5 (3.00%)
Completed school education	105 (37.20%)	50 (29.90%)
Professional qualification	7 (2.50%)	5 (3.00%)
Bachelor's degree	138 (48.90%)	78 (46.70%)
Master's degree	28 (9.90%)	24 (14.40%)
PhD	2 (0.70%)	4 (2.4%)

*Note.* MENA = Middle East and North Africa.

### Design and Materials

The quantitative analysis featured a 3 × 3 between-subjects design with the factors: perpetrator justification (no justification, sexual deprivation, jealousy) and victim resistance (verbal, verbal and physical, and passive). Originally, researchers designed nine different MR scenarios. All vignettes include a nonconsensual husband-to-wife intercourse scenario. Intimate partner violence is often researched using a vignette-based design (Monson et al., 2000; Munge et al., 2007; Persson & Dhingra, 2022). A common shortcoming of vignettes being used is that MR scenarios offered to subjects often paint MR as including a great deal of physical violence (Monson et al., 2000), which does not necessarily reflect the true nature of this type of violence. On top of that, most studies have been conducted on women who have sought help at rape crisis centers or battered women's shelters. Therefore, their findings may not be generalizable to other victims of MR (Martin et al., 2007). Therefore, vignettes used for this study have a more subtle language, do not include any information about physical aggression and do not use the word “rape” to avoid any potential bias. It was important for researchers not to prime participants with any information on the level of violence, quality of marital relationships, and physical or personality characteristics of the participants. To standardize the vignettes, only one sentence was changed in each scenario.

While these vignettes have been created by the researchers, they are inspired by previous vignette-based research on domestic violence and rape myths (Ewoldt et al., 2000; Monson et al., 2000; Munge et al., 2007). The description of the MR scene is much the same as the way it was described by Munge et al. (2007), except for the way different levels of resistance were manipulated.

Vignettes were translated from English to Arabic for those participants from the MENA region who would prefer the survey to be answered in Arabic. Back translations were used to ensure reliability. As this was a pilot study and it intended to see which jealousy manipulation works better for participants from MENA and South Asia, additional vignettes were added, and overall, 15 different vignettes were used. These additional vignettes were created because of the sensitivity of the issue in both regions. Therefore, it was decided that original manipulation (prior to the rape, a wife danced with a stranger at a party) might be perceived as a too Westernized behavior, in other words, a foreign and out-group pattern. Thus, we have added two more ways how jealousy might be manipulated: (a) a wife only talked to a stranger at the party but did not dance; (b) a wife comes home late from work, where her boss is a man. However, after running the analysis, no statistically significant differences in RS, VB, and PS were detected between different manipulations of jealousy. Therefore, in this study, different manipulations of jealousy will be analyzed together and called “jealousy condition.”

After reading an MR scenario, a manipulation check was administered through a brief attention test to ensure the participant read the vignette carefully. These questions included generic details and did not prime the participants in any way. Participants who were unable to answer at least two of the three questions correctly were excluded from the analysis. However, among those participants who did not withdraw before submitting the survey, everyone answered at least two questions correctly.

Then participants answered follow-up questions on PS of the scenario (five items; e.g., “This was a violent incident”), VB attribution (four items; e.g., “How much did ‘Wife's name’ enjoy this situation?), and RS attribution (four items; e.g., “To what degree were ‘Husband's name’ actions a violation of ‘Wife's’ rights?”). For each item, participants were presented with a 7-point rating scale from *strongly disagree or minimal* (1) to *strongly agree or extreme* (7) (Monson et al., 2000; Yamawaki et al., 2009).

## Results

All scales showed acceptable to excellent internal consistency for the whole sample: .91 for RS, .79 for VB, and .90 for PS. Similar results were obtained when checking for two separate subsamples. For MENA region participants, all scales showed good to excellent internal consistency for the whole sample: .92 for RS, .86 for VB, and .91 for PS. In the South Asian subsample, two scales showed good and excellent internal consistency: .87 for RS and .90 for PS. Cronbach's α for VB was .57.

In the next step, we administrated three factorial ANOVAs to explore general patterns of RS, VB, and PS of rape in the whole sample. Tables 3–5 summarize means and standard deviations for RS, VB, and PS as functions of justification and level of resistance.

**Table 3.** Preliminary Analysis for the Whole Sample.

	Rape support		
	No justification	Jealousy	Sexual deprivation
Passive resistance	12.31 (6.76)	11.00 (6.20)	12.61 (7.68)
Verbal resistance	8.87 (5.28)	9.01 (5.55)	10.85 (7.15)
Verbal and physical resistance	9.03 (5.68)	8.82 (5.71)	10.54 (6.60)

*Note.* Means and standard deviation of rape support as a function of justification and level of resistance.

**Table 4.** Preliminary Analysis for the Whole Sample.

	Victim blaming		
	No justification	Jealousy	Sexual deprivation
Passive resistance	9.31 (4.22)	9.62 (5.07)	10.61 (6.97)
Verbal resistance	9.67 (6.50)	10.20 (6.70)	11.62 (7.52)
Verbal and physical resistance	8.68 (4.45)	9.11 (5.88)	11.71 (6.81)

*Note.* Means and standard deviation of victim blaming as a function of justification and level of resistance.

**Table 5.** Preliminary Analysis for the Whole Sample.

	Perceived seriousness		
	No justification	Jealousy	Sexual deprivation
Passive resistance	22.83 (8.20)	23.62 (9.16)	22.45 (9.88)
Verbal resistance	26.93 (5.80)	27.12 (6.84)	25.17 (7.79)
Verbal and physical resistance	27.46 (7.02)	28.68 (7.19)	24.66 (9.00)

*Note.* Means and standard deviation of perceived seriousness of rape as a function of justification and level of resistance.

Across scenarios, the greatest RS was received in the scenarios with passive resistance compared to verbal resistance or verbal and physical resistance. When paired with different types of justification, the following pattern emerged: the greatest level of RS was found in the passive resistance plus sexual deprivation scenario (*M* = 12.61, *SD* = 7.68), RS was a bit less in the passive resistance, and no justification (*M* = 12.31, *SD* = 6.76), and the least RS was found in the passive and jealousy scenario (*M* = 10.99, *SD* = 6.20). Both the effect of type of justification, *F* (2, 555) = 4.22, *p* = .015, η² = .015, and level of resistance, *F* (2, 555) = 7.94, *p* < .01, η² = .028, were found to be significant. Post-hoc analyses using a *t*-test (Bonferroni-corrected pfwe = .05) indicated that RS was greater for sexual deprivation than for jealousy (*X*_diff_ = 1.76, *p* = .025), as for the level of resistance, RS was greater for passive resistance in comparison to verbal resistance (*X*_diff_ = 2.11, *p* = .003) and verbal and physical resistance (*X*_diff_ = 2.64, *p* < .001).

As for VB, the greatest VB was found in sexual deprivation and verbal and physical resistance scenario (*M* = 11.71, *SD* = 6.81), followed by sexual deprivation and verbal resistance (*M* = 11.62, *SD* = 7.52), whereas the smallest VB was obtained in no justification and verbal and physical level of resistance (*M* = 8.68, *SD* = 4.45). For VB, only the type of justification was found to have a significant effect, *F* (2, 549) = 3.95, *p* = .02, η² = .014. Post-hoc analyses using a *t*-test (Bonferroni-corrected pfwe = .05) indicated that VB was greater when sexual deprivation was offered as a justification in comparison to no justification (*X*_diff_ = 2.17, *p* = .028), and in comparison, to jealousy as a justification (*X*_diff_ = 1.72, *p* = .027).

PS was the greatest in jealousy and verbal and physical resistance scenarios (*M* = 28.68, *SD* = 7.19), and the rape was perceived as less severe in the sexual deprivation passive resistance scenario (*M* = 22.45, *SD* = 9.88). Both the effect of type of justification, *F* (2, 554) = 3.79, *p* = .023, η² = .013) and level of resistance, *F* (2, 554) = 9.97, *p* < .01, η² = .035, were found to be significant. Post-hoc analyses using a *t*-test (Bonferroni-corrected pfwe = .05) indicated that PS was greater for sexual jealousy than for sexual deprivation (*X*_diff_ = 2.14, *p* < .05), as for the level of resistance, PS was greater for verbal resistance in comparison to passive resistance (*X*_diff_ = 3.38, *p* < .01), and for verbal and physical resistance in comparison to passive resistance (*X*_diff_ = 4.29, *p* < .001).

### Analysis of the Effects of Justification and Level of Resistance on RS, VB, and PS in South Asian Sample

We administrated three factorial ANOVAs to explore the effects of justification and level of resistance on RS, VB, and PS of rape in the South Asian subsample (see Tables 6–8 for means and standard deviations).

**Table 6.** Analysis in South Asian and MENA Samples.

	Rape support		
	No justification	Jealousy	Sexual deprivation
South Asian sample			
Passive resistance	12 (6.08)	10.28 (5.60)	11.46 (7.31)
Verbal resistance	6.38 (2.33)	8.32 (4.50)	7.36 (3.46)
Verbal and physical resistance	7.95 (3.69)	6.52 (3.49)	8.33 (3.03)
MENA sample			
Passive resistance	13.73 (7.56)	12.47 (7.50)	15.54 (8.49)
Verbal resistance	12.78 (6.83)	12.61 (7.51)	17.85 (7.97)
Verbal and physical resistance	13.71 (9.18)	12.00 (7.67)	13.53 (8.57)

*Note.* MENA = Middle East and North Africa.

Means and standard deviation of rape support as a function of justification and level of resistance.

**Table 7.** Analysis in South Asian and MENA Samples.

	Victim blaming		
	No justification	Jealousy	Sexual deprivation
South Asian sample			
Passive resistance	8.94 (3.73)	8.52 (4.17)	8.46 (4.46)
Verbal resistance	9.44 (5.70)	9.02 (4.88)	8.38 (3.83)
Verbal and physical resistance	7.16 (2.67)	8.18 (3.58)	9.25 (3.84)
MENA sample			
Passive resistance	10 (5.28)	12.08 (6.37)	14.08 (8.62)
Verbal resistance	12.00 (8.77)	14.71 (9.20)	18.23 (8.82)
Verbal and physical resistance	12.86 (6.79)	12.10 (8.73)	14.13 (8.47)

*Note.* MENA = Middle East and North Africa.

Means and standard deviation of victim blaming as a function of justification and level of resistance.

**Table 8.** Analysis in South Asian and MENA Samples.

	Perceived seriousness		
	No justification	Jealousy	Sexual deprivation
South Asian sample			
Passive resistance	22.71 (8.05)	24.34 (8.88)	22.38 (10.94)
Verbal resistance	29.88 (2.92)	27.39 (6.43)	28.59 (4.51)
Verbal and physical resistance	27.11 (7.67)	29.56 (5.56)	26.92 (6.72)
MENA sample			
Passive resistance	21.33 (8.82)	23.03 (10.67)	19.77 (9.33)
Verbal resistance	22.89 (6.85)	24.81 (8.78)	18.46 (8.83)
Verbal and physical resistance	27.29 (6.29)	25.83 (9.41)	21.07 (10.40)

*Note.* MENA = Middle East and North Africa.

Means and standard deviation of perceived seriousness of rape as a function of justification and level of resistance.

Participants from the South Asia showed the greatest RS in scenario with no justification and passive resistance (*M* = 12.00, *SD* = 6.08), followed by sexual deprivation and passive resistance scenario (*M* = 11.46, *SD* = 7.31), the least support was received by the scenario with no justification and verbal resistance (*M* = 6.38, *SD* = 2.33). Only the level of resistance was found to have a significant effect, *F* (2, 274) = 13.93, *p* < .01, η² = .092. Post-hoc analyses using a *t*-test (Bonferroni-corrected pfwe = .05) showed that *r* was greater for passive resistance than for verbal (*X*_diff_ = 2.98, *p* < .01) and verbal and physical resistance (*X*_diff_ = 3.71, *p* < .01).

As for VB in the South Asian subsample, the greatest was found in the no justification and verbal level of resistance scenario (*M* = 9.44, *SD* = 5.70) and the smallest in the no justification and verbal and physical resistance scenario (*M* = 7.16, *SD* = 2.67). However, these differences are not statistically significant as no main effects of type of jealousy or level of resistance were found.

PS was the greatest in the no justification and verbal resistance scenario (*M* = 29.88, *SD* = 2.92) followed by the jealousy and verbal and physical resistance scenario (*M* = 29.56, *SD* = 5.56), whereas the scenario with sexual deprivation offered as a justification and passive resistance was seen as the least serious (*M* = 22.38, *SD* = 10.94). Similar to RS, the level of resistance was found to have a significant effect, *F* (2, 273) = 10.87, *p* < .01, η² = .074. Post-hoc analyses using a *t*-test (Bonferroni-corrected pfwe = .05) indicated that PS was greater for verbal resistance than for passive resistance (*X*_diff_ = 4.31, *p* < .01) and for verbal and physical compared to passive resistance (*X*_diff_ = 4.95, *p* < .01).

### Analysis of the Effects of Justification and Level of Resistance on RS, VB, and PS in the MENA Region Subsample

To examine the effects of justification and level of resistance on RS, VB, and PS of rape in the MENA region subsample, we administrated three factorial ANOVAs (see Tables 6–8 for means and standard deviations).

Participants from the MENA region showed the greatest RS in a scenario with sexual deprivation and verbal level of resistance (*M* = 17.85, *SD* = 7.97), followed by sexual deprivation and passive resistance scenario (*M* = 15.54, *SD* = 8.49), the smallest support was received by the scenario with jealousy and verbal and physical level of resistance (*M* = 12.00, *SD* = 7.66). The type of justification was found to be marginally significant in RS, *F* (2, 158) = 2.52, *p* = .08, η² = .031.

As for VB, participants from the MENA tended to blame the victim the most in sexual deprivation and verbal level of resistance scenario (*M* = 18.23, *SD* = 8.82) and the smallest level of VB was found in no justification and passive resistance scenario (*M* = 10.00, *SD* = 5.28). However, these differences failed to reach the level of statistical significance.

PS was the greatest in no justification and verbal and physical resistance scenario (*M* = 27.29, *SD* = 6.29) followed by jealousy and verbal and physical resistance scenario (*M* = 25.83, *SD* = 9.41), whereas scenario with sexual deprivation offered as a justification and verbal resistance was seen as the least serious (*M* = 18.46, *SD* = 8.83). The type of justification was found to have a significant effect on PS, *F* (2, 157) = 3.74, *p* = .026, η² = .045. Post-hoc analyses using a *t*-test (Bonferroni-corrected pfwe = .05) indicated that PS was greater for jealousy than for sexual deprivation (*X*_diff_ = 4.58, *p* < .05).

### Comparison of the Levels of RP, VB, and PS Between Participants From the MENA Region and South Asia

To examine cross-cultural differences in RS, VB, and PS, several independent-samples *t*-test were administrated. The results indicated that participants from the MENA region (*M* = 13.35, *SD* = 7.77) demonstrated greater RS than participants from South Asia (*M* = 8.52, *SD* = 4.86), *t*(448) = –8.111, *p* < .01 95% CI [–5.99, −3.66]). VB was also found to be greater in the MENA subsample (*M* = 13.20, *SD* = 8.04), than in the South Asian subsample (*M* = 8.55, *SD* = 4.15), (*t*(443) = –8.04, *p* < .01 [–5.79, −3.52]). In relation to PS of rape, South Asian participants had a greater tendency to see it as a serious assault (*M* = 26.87, *SD* = 7.40), than participants from the MENA region (*M* = 23.04, *SD* = 9.48), (*t*(446) = 4.76, *p* < .01 [2.25, 5.41]).

## Discussion

The current study explored how the level of resistance, as well as different kinds of justification, such as the perpetrator's sexual deprivation and jealousy, impact the perception of MR. The results of this study suggest that the type of justification significantly predicts VB, RS, and PS of MR, whereas the level of resistance predicts RS and PS but not VB.

When samples were combined, RS and VB were found to be greater when sexual deprivation was offered as a justification in comparison to jealousy and no justification. On the other hand, PS was greater in jealousy conditions. Because the influence of sexual deprivation on the perception of MR has not been previously studied, it is difficult to compare this finding with other research. However, this result is consistent with findings regarding sexual objectification, which changes the way people view women by reducing them to sexual objects and increasing VB (Loughnan et al., 2013). Although previous research did not address how jealousy might impact the perception of MR, it suggests that jealousy is strongly related to intimate partner violence in general (Pichon et al., 2020) and might be one of the reasons for MR. Husbands who perpetrate MR may engage in this behavior because they are more jealous of their wives. However, wives who have been subject to MR were no more likely than other abused women to be having extramarital relationships (Frieze, 1983; Gelles, 1977). Studies on dating violence discuss how physical violence seemed to be more justifiable following a sexual betrayal, especially the hitting of a male partner by a betrayed woman (Forbes et al., 2005). When it comes to the influence of fidelity status on the perception of MR, wives who are unfaithful to their husbands are assigned greater responsibility for the MR (Munge et al., 2007). Thus, considering this study manipulated jealousy very subtly and did not manipulate the fidelity status or imply that the wife was truly unfaithful, participants might not have seen this manipulation as serious enough to justify rape.

As for the level of resistance, RS was greater for passive resistance in comparison to verbal resistance and verbal and physical resistance, and the event was perceived as more serious in verbal resistance and in verbal and physical resistance conditions in comparison to passive resistance. However, there were no differences between verbal resistance and verbal physical resistance. These findings are in line with previous studies on acquaintance rape, which found that relative to no resistance, verbal and physical strategies by the victim predicted higher levels of victim credibility, perpetrator culpability, and perpetrator guilt, as well as lower levels of victim culpability and perceived victim pleasure (Angelone et al., 2015). At the same time, studies, where relationship status is not specified, failed to detect the effect of resistance on the ratings of victims’ responsibility (Sims et al., 2007). Thus, the relationship status plays an important role, and future research might look into the effect of different levels of resistance based on the relationship status. It is important to note that both the data from this study and the studies cited relate primarily to female victims, as the few studies conducted on male victims give a very different picture. For example, in a study of the role of victim sexuality and level of resistance in VB, male participants blamed a gay victim more when he fought back against his attacker but, conversely, blamed a heterosexual victim when he did not fight back (Davies et al., 2008).

The results were slightly different for the two chosen regions, which indirectly supports the necessity to examine the topic of MR in different cultures and not overgeneralize findings acquired in one region only. The type of justification was not a significant predictor of VB, RS, or PS of rape. However, the level of resistance impacted both RS and PS. It was found that South Asian participants demonstrated greater RS in the passive resistance conditions and perceived events as more serious in the case of verbal resistance and verbal, physical resistance in comparison to passive resistance. Interestingly, the level of resistance was not a significant predictor of any variables in the MENA sample, but the type of justification was significant for PS and marginally significant for RS. Just like for the overall sample, sexual deprivation was associated with greater RS and jealousy with greater PS. As mentioned in the introduction to this article, there are no studies of perceptions of MR conducted in the MENA region, so we cannot compare the data from this study with those from similar studies. In the case of South Asia, as noted, there has been a gradual emergence of scholarly interest in the region on the topic of MR (e.g., Agarwal et al., 2022; Banerjee & Rao, 2022; Damania & Singh, 2022; Deosthali et al., 2022; Rao et al., 2022; Saleem, 2021). However, existing studies have not examined the variables discussed in this study and, therefore, cannot serve as a basis for comparison.

As predicted, while comparing the answers from participants from the MENA region and South Asia, it was found that participants from the MENA region exhibited greater RS and VB than participants from South Asia. In contrast, participants from South Asia demonstrated higher levels of PS. These differences may reflect both the general state of human rights in a country and uniquely indicate a woman's place. Indeed, according to the Human Rights and Rule of Law, South Asian countries have a more favorable human rights rating index (TheGlobalEconomy.com at https://www.theglobaleconomy.com). Still, the Global Gender Gap Index is not as clear-cut as many MENA countries have a higher rating than South Asian countries (World Economic Forum. Global Gender Gap Report, 2022). Therefore, the differences may be due to cultural or religious differences.

This study provides several contributions, being one of the first, if not the first one, exploring factors such as jealousy, sexual deprivation, and level of resistance in South Asian and MENA region communities. Studying underrepresented samples is a key strength of the study, allowing for a nuanced understanding of the perceptions of MR, which in turn might be used later for tailored interventions to educate people about MR and to prevent it. Moreover, to our knowledge, it is also the first study looking into the effect of sexual deprivation.

At the same time, several limitations should be noted. First, the relatively low number of participants limits the statistical power and generalizability of the findings (Button et al., 2013). However, the sample sizes used in this study—116 participants in one region and 451 in another—fall within acceptable ranges for exploratory cross-cultural research and survey-based designs (Dattalo, 2009; van de Vijver & Leung, 1997). While larger and more representative samples would strengthen generalizability, the current sample sizes are adequate for identifying meaningful patterns and generating hypotheses for future research.

Additionally, the use of convenience and snowball sampling introduces a degree of sampling bias, which may have skewed the sample toward individuals who are more educated, technologically literate, and socially connected (Etikan et al., 2016). Such participants are also more likely to have been exposed to global discourses on gender equality and violence, which may have influenced their responses and limited the ability to capture more traditional or conservative viewpoints prevalent in some rural or marginalized communities. However, we do not believe this posed a significant concern in the present study. This method is frequently used in socioculturally sensitive, cross-cultural research when direct access is difficult—for example, in studies on domestic violence within Muslim communities (Shohel et al., 2015). In addition, snowball sampling is recognized as one of the most feasible and ethically sound methods for engaging hard-to-reach or vulnerable populations, particularly when participants are hidden due to linguistic, cultural, or political barriers (Sadler et al., 2010). Therefore, while not ideal, this sampling strategy was appropriate and effective given the exploratory nature of our study and the context-specific access limitations.

Another limitation relates to the heterogeneity of the MENA sample. The MENA region includes a broad mix of countries that may differ significantly in their laws, cultures, and gender norms. In some contexts, MR may be legally recognized, while in others it is overlooked or even justified based on prevailing interpretations of religion or tradition. Therefore, collapsing participants from multiple MENA countries into one category may obscure meaningful intraregional differences (Joseph & Naǧmābādī, 2003). This limits the generalizability of the findings and potentially masks unique cultural or legal influences on attitudes toward MR. However, given the lack of prior research on this topic within the region, this study offers a valuable initial contribution that can serve as a foundation for more nuanced, country-specific investigations in the future.

Similarly, the religious, cultural, and legal diversity within the MENA communities themselves further complicates interpretation. While the study refers to the region broadly, countries within MENA operate under different family laws—ranging from civil to Sharia-based systems—with varying implications for marital rights, consent, and female agency (Hajjar, 2004). Religious affiliation (e.g., Sunni, Shia, Christian, and Druze) and levels of secularism can also drastically shape gender relations and interpretations of spousal duty and resistance. Therefore, attitudes toward MR in one MENA subpopulation may not be representative of others within the same region. Nevertheless, by examining the region as a whole, this study provides important initial insights into a highly complex and understudied area, laying the groundwork for more detailed, context-specific research to follow.

Finally, the study did not gather information about whether participants lived in urban or rural areas. Rural participants may hold more traditional beliefs about marriage and gender roles than their urban counterparts, and these differences could significantly impact perceptions of MR. This limitation is common in survey research, as rural populations are often harder to reach due to limited internet access and lower participation rates (Salemink, Strijker & Bosworth, 2017). Despite this challenge, the study delivers meaningful initial insights into perceptions of MR across diverse contexts and underscores the need for future studies to specifically target rural communities.

It could be concluded that overall MR continues to be somewhat justified in cases where the victim does not demonstrate behaviors expected from her or behaves in ways that do not fit the cultural perception of women or marriage in general. Interestingly, contrary to previous research, jealousy was not found to be an important predictor that altered the perception of MR, but sexual deprivation seems to play an important role in this perception. That implies that the concept of marital duty is still deeply internalized and that society does not see female sexuality as something belonging to a woman but rather as being owned and controlled by the man, especially in marriage. This study also supported the idea that the level of resistance is an important factor to consider, but as discussed above, the relationship status and gender of the victim need further investigation. Passive resistance was associated with the greatest VB and RS, which is very concerning, as this type of resistance might also mean a chronic nature of the event and be a sign of learned helplessness and lack of access to legal and social recourse. Therefore, there is a need for interventions which will address the complex nature of couple dynamics and the chronic nature of domestic abuse in general and MR in particular.
